# Practical Medicine

**Published:** 1877-03

**Authors:** 


1. *The Curved Line of Pleuritic Effusion.* ELLIS. (*Boston Med. and Surg Jour.*, Dec. 14, 1876.)

Five cases of effusion into the pleural cavity, each having the curved lines which limited the dullness superiorly, are reported by Dr. Ellis.

The Dr. very ingeniously elucidates, by diagrams, the extent of effusion following the curved lines. He also reports a case where the flatness extended to the right nipple and around the chest, varying with a change of position. This case is the first that attracted the attention of the author to the peculiar form of dullness in pleurisy. He claims to have established the fact that the dullness in pleuritic effusion is most obvious in the sub-axillary region.

Nov. 10, 1873. A lad of nineteen had an attack of pleurisy. There was dullness over the whole right side. Nov. 25th. Absorption became manifest through a change in the line of dullness. The line followed a curve, beginning at the junction of the sternum with the costal cartilage of the fifth rib of the right side, extending obliquely upward to and crossing the third inter-costal space, thence obliquely downward and inward to the fifth rib of right side posteriorly, thence dropping almost parallel with the vertebral column until it arrived opposite the eighth dorsal vertebra, where it terminated at almost right angles with the column. Dec. 28th. The curved line of dullness in part changed, commencing anteriorly as before, passing almost horizontally to the sixth inter-costal space, crossing it, then following parallel with the same line of curvature as that described above, to the ninth dorsal vertebra.

An Englishman, on Oct. 3, 1873, was attacked with pleurisy. The right side of chest was flat, except the top of the shoulder and clavicular region. The heart was pushed to the left. The curved line of dullness, beginning at the second rib posteriorly, passing upward and outward and crossing the first rib of the right side, thence taking a transverse direction across the chest until it gained the junction of the first costal cartilage with the sternum, taking thence an oblique course downward and outward, it passed about two inches to the outside of the nipple to the fifth rib of left side below, where it curved upon itself, becoming lost at the articulation of the costal cartilage with the sixth rib. Normal respiration was heard above and slightly below the line of dullness, and over the back soft bronchial respiration. Nov. 2d. The line of dullness was found to extend from the eighth dorsal vertebra upward and outward in shape of the italic letter *f*, crossing the fourth rib of right side to the third rib of left side, thence curving parallel with the one above described, though just inside of the left nipple, terminating at the union of the fifth rib of left side with the sternum. Nov. 3d. The dullness had not changed; but the râles, sibilant and sonorous, had disappeared, and the mucous râles were not so abundant. Normal respiration was detected, following the line somewhat as indicated, and about two inches outward, increasing toward the upper part. The other cases as reported were similar, each showing the curved lines of dullness.

Dr. Damoisean, in 1853, observed the lines of dullness in pleuritic patients, and substantiated his discovery by means of the trocar. In the patients who were recovering from effusion he noticed these facts, namely: "While the flatness persisted in the sub-axillary region on a level with and beneath the lower angle of the scapula, there was resonance in the lower part of the vertebral groove. *Secondly*, the flatness always disappeared last from the lowest sub-axillary region." He also stated that when the dulness reached a point about two and three-fourths inches above the nipple, the line was nearly horizonal; beneath this level it was a parabolic curve, the curvature increasing until it became elliptical in the lower and lateral parts of the hypochondrium.

The author urges close attention to the lowest sub-axillary region, believing with D. that the presence of an ounce of fluid can be detected there. Just how the curved line is formed Dr. E. fails to tell us; of the value of it he thinks there can be no doubt. W. F. L.

2. *A Case of Intermittent Pneumonia.* HILDEBRANDT. (*Deutsche Med. Wochenschr.*, 1876, No. 49.)

On Nov. 13th, a soldier was admitted to the hospital complaining of pain in the right side, chills and cough. He coughed considerably, and threw up the characteristic pneumonic sputa; crepitant râles over the lower posterior portion of the right lung; no dullness on percussion. Temp. 39.5° C. Nov. 14th A. M. Respiration easy and free of pain and cough; very few râles in the right side; temp. 37.5°; slight enlargment of the spleen. At 3 P. M. a violent chill followed by headache; cough with pneumonic sputa; crepitant râles and a temperature of 45.5°. Nov. 15 A. M. Temp. 37.5°; patient felt quite well, though he coughed some and the crepitant râles had not entirely disappeared. Instead of sodæ nitras he now took thirty grains of quinine. The fever did not return; the evening temperature was 37.5°. Nov. 16 A. M. Temp. 36.5°; sputa muco-purulent and scanty. Nov. 19. Patient was discharged and has entered on duty.

3. *Maternal Impressions Affecting the Fœtus.* (*Philadelphia Med. Times* Dec., 1876.)

In the cases of naevi materni, reported by Dr. E. Seguin, occurs one in his own family. The wife of a physician longed for a dish of ham, her husband thinking this a good opportunity to try the reality of the doctrine of maternal impressions, refused the covetous dish. A girl was born with a mark not unlike the appearance of cooked ham on the back of the head and on the legs. He attended a lady who, during her pregnancy, was so exercised for the safety of her husband engaged in war that she gave birth to an idiotic child; all the other children (6) were above mediocrity.

Another lady—very refined—drank daily a quart of brandy while carrying her fourth child; experienced no uncomfortable symptoms from it; gave birth to an idiotic boy.

A lady came out over-heated from a ball room; her baby three months old taking the breast, was seized with spasms two hours after, and since, is a confirmed idiot and epileptic. In a moment of great anxiety a lady jumped into a carriage with her child, a girl of fifteen months, whose intellect so far was very good. During a journey of twenty miles the child took the breast once, vomiting before arriving at the destination; acute fever followed. The child became a cripple and an idiot. A tramp called at the residence of a farmer for a pot of milk and bread; the wife seeing that he had no left hand re-entered the room crying, " My child will be born with but one hand," and in two months later it so happened. A gentleman lying sick in a country house at night, was attended by his wife, they being alone in the house. She saw somebody wrapped in a sheet trying to effect an entrance; being unprotected she cried out, piled furniture against the door and succeeded in repulsing the intruder. She gave birth, soon after, to a healthy male child, who, at the hour at which the struggle occurred, would scream out every night as if in distress, at other times was good natured. This habit disappeared when taken from the breast. W. F. L.

4. *Cerebral Exhaustion.* SCHWEIG. (*The Med. Record*, 1876, No. 313.)

The chief predisposing cause of cerebral exhaustion, says the author, is the lack of stamina or staying power, superinduced by too long continued thought or hard study. The avocation of a man is an important factor in the genesis of the disease; physicians, lawyers and inventors furnish the largest proportion of this class of patients. The middle aged, young and old are somewhat exempt. Dr. S. does not believe that the female is less predisposed than the male, but the avocations that distinguish the sexes accounts for the small quota of the disease furnished by women. " Abuse of the mental faculties " is a direct cause of cerebral neurasthenia; such as depressing emotions resulting from family or business troubles, sickness, etc. These causes combined render the brain less competent to resist taxations that, under ordinary circumstances, it might sustain. Of the pathology he thinks little can be said, but none appears more rational than the theory of nutritial changes. Of the symptoms the most prominent is a disinclination for mental labor. If this feeling is only temporary, and an attempt made to perform brain work, a brief application will exhaust the powers of thought and bring on confusion of ideas. In many cases the memory is weakened, in others there is no perceptible change.

Insomnia is present from the beginning. Organs over which the pneumogastric nerve presides are more disturbed than all the others. Disturbances of the heart's action, stomach, liver, intestinal canal, with vasomotor irregularity are common. Cerebral nerves, other than the one described, may in turn become involved; in proof of which we have neuralgia of varied form and intensity.

A mistake in diagnosis is not uncommon; the symptoms due to functional disturbance of the pneumogastric may be looked upon as primary conditions, and result in the loss of opportunity for rapid and decisive treatment. Prognosis will depend not only on the severity of the case, but upon the hereditary disposition, and cheifly, perhaps, upon the ability to follow strictly the treatment. The paramount condition for treatment "is rest for the exhausted brain;" not a temporary rest, but absolute abstinence from all mental labor throughout the entire treatment.

Dr. S.'s method of treatment consists in general galvanization of the brain. To do this he makes use of the galvanic bath. In doing so every organ of the body comes under the electric influence; dyspeptic symptoms, torpor of the liver, cardiac derangement, etc., as a rule are ameliorated. In conjunction with this line of treatment he makes use of the faradic current as a prophylactic, and by evoking capillary stasis a powerful auxilary is furnished the galvanic current, and better results obtained. A good nourishing diet, with either phosphorus or cod-liver oil is necessary. As regards the administration of the baths, the author admonishes against the use of strong currents at the beginning; the faradic may be used with the bath currents from the first, although the faradic current is only necessary where there is a sub-paralytic condition. The galvanic should take precedence of the faradic current and not be employed more than ten minutes. The bath should not be continued long enough to fatigue the patient. W. F. L.

5. *Tetanus Successfully Treated by Calabar Bean.* GRANT. (*The Med. Record*, Dec., 1876.)

Many of the failures attending the administration of Calabar bean for tetanus have been due either to a poor preparation of the drug or insufficient doses. A German having tetanic spasms was first given of the tincture 10 drops every hour; on the subsequent day 20 drops, increasing to 30 every hour. Patient now improving, it was reduced to 30 drops three times daily; when suddenly the spasms returning, the following was given:

℞ Extract. Physostig.................................... grs. xxxij
Alcohol. dil.................................................... ℥j
M. S. 45 drops every two hours.

The spasms soon subsiding, the patient was given the dose three times daily. The afternoon of the next day the attacks again returned; the dose was now increased to 60 drops every hour for two days; spasms having now disappeared patient was put on 30 drops three times a day. From this date there was no return of spasms. The only physiological action of the agent was contraction of the pupils and slight delirium at times.

W. F. L.

6. *Case of Peliosis Rheumatica.* TAYLOR. (*The Am. Pract.* Dec., 1876.)

A married woman, aged 48, for several years had poor health; was, in

January last, taken with acute pain in the middle of the spine, extending obliquely around the body, and terminating in the left groin. An eruption of herpes zoster, in irregular patches and in its different stages, followed the track of pain, terminating in about two weeks. At the end of the fourth week the pain shifted its location to the thigh and leg, following the great ischiatic and posterior tibial nerves, settling in the foot.

About the fourth or fifth day the foot and ankle became swollen, pitting on pressure, and being painful to the touch. The next morning the anterior portion of the foot and lower half of leg were covered by dark red spots, more numerous over the ankle joint, and from the size of a shot to that of a silver dime, and not projecting above the surface. They retained this color for two days, then changing from dark to brown, then to yellow fading away without desquamation. The eruption lasted ten days, during which time pain and swelling was less severe. At the end of the fourteenth day she had a second attack, followed, each after a similar interval, by a third and fourth one, and each running nearly the same course, all the difference being that the last attack implicated the whole leg, and continued for months with no cessation. Treatment did no good. Finally, on the approach of autumnal weather, seven months after the first attack, a change for the better occurred which continued until she recovered. A loud systolic murmur was detected, with its greatest intensity at the apex.

The author inquires why the periodicity that was present did not yield to quinia and arsenic if malaria was the cause; and why the periodicity if the disease was due to detached fibrinous masses floating in the small arteries and capillaries, resulting from heart disease, and thus poisoning the blood. W. F. L.

7. *A Case of Intestinal Obstruction of Eighteen Weeks.* BLAKE. (*The Boston Med. and Surg. Jour.*, Nov. 23, 1876.)

A man 46 years of age, strongly developed, weighing 180 pounds, mechanic, of good habits, and health usually fair up to his last sickness in November last; had reverses in business which created a good deal of mental excitement and derangement of digestion. Feb. 29th. Dr. B. was called and found the patient had taken freely of cathartics followed by no fecal evacuations, but by a discharge of glairy mucus mixed with blood, accompanied by tenesmus and pain. Examination revealed no tumor, nor pain, nor tenderness on pressure at any point. During the next week no change in patient's condition; soon afterward followed tympanitis, vomiting and hiccough. Emaciation soon supervened, and before death he was a "living skeleton." June 27th. Bowels moved spontaneously every hour, without power of resistance; the matter evacuated having a tarry look. The quantity the first day was about a gallon; the number of operations from day to day gradually diminished until death. Treatment consisted of injections, calomel pill, followed by castor oil and morphine to relieve pain. Fifth day of illness; bowels had become swollen; enemata of oil of turpentine, castor oil, and a gallon of warm water were administered, giving immediate relief. After four days this treatment failed.

Tenth day, tympanitic distention endangered life, pushing the liver as high as the nipple, the heart pulsations appearing between second and third ribs. Inflation was made without relief. The aspirator was now used; Patain's smallest needle being introduced an inch above the umbilicus, and the flatus pumped off precisely as fluid would be. The tense condition disappeared, the lungs expanded, and the pulse fell from 140 to 96, wonderfully improving the patient's condition. An attempt to move the bowels was now made and failed. A trial at inflation was again made without giving relief. All methods of treatment now being abandoned, nourishing diet per rectum, morphine and the aspirator were alone used; the latter freely without lighting up any inflammation or giving patient any pain. The most wonderful feature of the treatment consisted in the free use of the aspirator. From the second week of sickness up to the time of death it was used daily, and quite frequently three times a day. It afforded so much relief that the patient would often call for it, telling the nurse where to puncture.

Dr. B. does not believe his patient could have survived many hours if the distention had not been relieved by the aspirator. Patient's life depended more on the use of the instrument than on morphine, nourishment and all the other treatment combined. W. F. L.

